# One-year impact of behavioural interventions on schistosomiasis-related knowledge, attitude and practices of primary schoolchildren in Pemba, Tanzania

**DOI:** 10.1186/s40249-024-01251-y

**Published:** 2024-11-13

**Authors:** Naomi C. Ndum, Lydia Trippler, Sarah O. Najim, Anisa S. Ali, Jan Hattendorf, Shaali M. Ame, Fatma Kabole, Jürg Utzinger, Said M. Ali, Stefanie Knopp

**Affiliations:** 1 Swiss Tropical and Public Health Institute, Allschwil, Switzerland; 2 University of Basel, Basel, Switzerland; 3 Public Health Laboratory-Ivo de Carneri, Pemba, United Republic of Tanzania; 4 Ministry of Health, Zanzibar, United Republic of Tanzania

**Keywords:** Attitude, Behaviour change communication, Control, Elimination, Intervention, Knowledge, Practices, Schistosomiasis, Tanzania

## Abstract

**Background:**

Elimination of schistosomiasis as a public health problem and interruption of transmission in selected areas are goals set by the World Health Organization for 2030. Behaviour change communication (BCC), coupled with other interventions, is considered an essential measure to reduce the transmission of *Schistosoma* infection. Focusing on elimination, we assessed the 1-year impact of BCC interventions on schistosomiasis-related knowledge, attitude and practices (KAP) of schoolchildren in hotspot schools versus low-prevalence schools that did not receive the interventions.

**Methods:**

School-based cross-sectional surveys were implemented in 16 schools on Pemba Island, Tanzania, in 2020 and 2022, respectively. The schistosomiasis-related KAP were assessed in children attending grades 3–5, using pre-tested questionnaires. Between the surveys, in 2021, children from hotspot schools were exposed to BCC interventions. The difference in mean knowledge and attitude scores, respectively, between schoolchildren from hotspot and low-prevalence schools during the survey in 2022 was determined with a linear mixed-effect model.

**Results:**

In the five hotspot schools that received BCC interventions, 315 children participated in the survey in 2020 and 349 in 2022. There was a 21.0% increase in children with moderate knowledge and a 13.8% decrease in no knowledge; a 8.3% increase in good attitude and a 19.2% decrease in poor attitude; 3.4% and 3.2% fewer children reported to use waterbodies for washing clothes or body, respectively. In the 11 low-prevalence schools without BCC interventions, 778 children participated in 2020 and 732 in 2022. The percentage of children with poor knowledge (56.4% and 63.1%) and poor attitude (55.3% and 53.1%) remained relatively stable from 2020 to 2022, but 4.9% and 3.0% less children reported to use waterbodies for washing clothes or their body, respectively. In 2022, the difference in mean knowledge scores was 0.8 [95% confidence interval (*CI*): 0.5−1.1] and the difference in mean attitude scores was 0.6 (95% *CI*: 0.4−0.7) between children in hotspot compared with low-prevalence schools.

**Conclusions:**

After one year of implementation, the BCC interventions markedly improved the KAP of exposed children. Complemented by improved access to clean water and sanitation, BCC holds promise to contribute successfully to the achievement of schistosomiasis control and elimination targets.

*Trial registration* ISRCTN, ISRCTN91431493. Registered 11 February. 2020, https://www.isrctn.com/ISRCTN91431493.

**Graphical Abstract:**

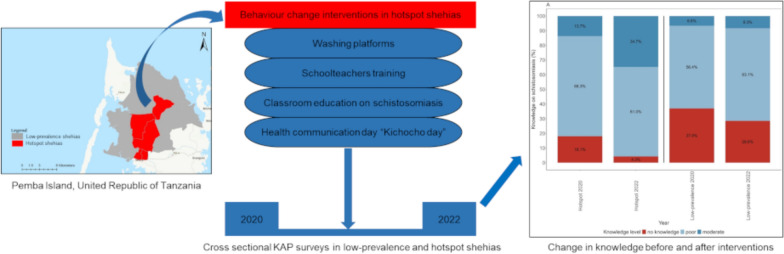

**Supplementary Information:**

The online version contains supplementary material available at 10.1186/s40249-024-01251-y.

## Background

Schistosomiasis is a neglected tropical disease (NTD) that is endemic in 78 countries [1, 2]. The global burden caused by schistosomiasis was estimated at 1.86 million disability adjusted life years in 2021 [1, 3]. The highest burden of schistosomiasis is found in Africa, where 91.3% of the people who require preventive chemotherapy live in 41 countries [4].

Over the past 20 years, efforts have been made to control the morbidity due to schistosomiasis in endemic areas [5–7]. The scale-up of preventive chemotherapy with praziquantel has resulted in a considerable decrease in schistosomiasis prevalence in sub-Saharan Africa [8]. In 2020, the World Health Organization (WHO) set the target to eliminate schistosomiasis as a public health problem by 2030 [9]. Areas that have achieved this goal are encouraged to move towards interruption of transmission [2]. In addition to preventive chemotherapy, WHO recommends water, sanitation and hygiene (WASH) interventions, environmental management, including water engineering and focal snail control with molluscicides, and behaviour change communication (BCC) as key measures to reduce *Schistosoma* transmission and to achieve elimination [2]. BCC is the strategic use of communications to deliver tailored messages to encourage individuals and communities to adopt healthier and more sustainable practices that might prevent illness [10]. As regards the control of NTDs, many programmes have moved progressively from preventive chemotherapy to a more holistic approach, including active participatory interventions like health education, community dialogue and behavioural interventions to improve and sustain intervention outcomes [11–14].

On the Zanzibar islands of the United Republic of Tanzania, health education and BCC have been important components of the efforts to control and eliminate urogenital schistosomiasis over the past century [5]. In the cluster-randomized trial of the Zanzibar Elimination of Schistosomiasis Transmission (ZEST) project (2012–2017), for example, one study arm assessed the benefit of adding BCC to preventive chemotherapy for schistosomiasis elimination in 15 randomized implementation units (IUs) [15, 16]. The BCC tools and approaches for the ZEST project were co-developed with the local communities in a human-centred design approach [17–19]. While the efforts of the ZEST project led to a remarkable decrease in the prevalence of *S. haematobium*, due to the very low overall infection numbers at the end of the trial, no statistically significant difference between the different intervention arms was identified [15, 16]. The SchistoBreak project, implemented from 2020 to 2024 in Pemba Island, aimed to assess new tools and strategies for breaking schistosomiasis transmission [20]. In the SchistoBreak project, the IUs were not randomized to receive interventions, but were stratified to interventions in line with small-scale *S. haematobium* prevalence thresholds. In hotspot IUs, a comprehensive intervention package consisting of preventive chemotherapy, focal snail control, BCC and small-scale WASH measures was implemented to further reduce prevalence. In low-prevalence IUs, surveillance-response interventions consisting of a test-treat-track-test-treat approach and reactive snail control were implemented to break transmission by a targeted use of resources [20, 21].

The study reported here is part of the SchistoBreak project and aimed to assess the 1-year impact of BCC interventions on schistosomiasis-related knowledge, attitude and practices (KAP) of children in hotspot schools versus the KAP of children in low-prevalence schools that did not receive the interventions.

## Methods

### Study area

The study was conducted on Pemba, one of the two main islands of the Zanzibar archipelago in the United Republic of Tanzania. Pemba has two regions (North and South) that are divided into four districts (i.e. Chake Chake, Micheweni, Mkoani and Wete), which are further subdivided into 129 small administrative units, known as shehias [22]. The SchistoBreak project was implemented from 2020 to 2024 in 20 shehias in the two northern districts of Pemba, Micheweni and Wete. In 2022, the 20 shehias had an estimated population of 95,000 [22]. A total of 81.5% of households in the Northern Region use improved sources of drinking water [23]. Piped water via taps and wells is available to 74.9% of household in this region [23]. For domestic activities and some recreational activities, the population of Pemba including children and adults rely mainly on the extraction of underground water from springs and wells, as tap water supply is often erratic [24, 25].

In 2020, there were 26 primary schools in the study area [26]. Furthermore, there were 239 Islamic schools (madrassas) located in the study area, which children attend in addition to primary or secondary schooling [17, 27]. The study presented here reports findings on the schistosomiasis-related KAP of children who attended the 16 main public primary schools in the study area and received BCC interventions in hotspot schools (comparing baseline results from late 2020 with follow-up results from early 2022).

### Study design and procedure

The SchistoBreak project was designed as an intervention study with repeated cross-sectional surveys [20]. The cross-sectional surveys served to assess the impact of interventions annually and to target interventions to the local micro-epidemiology. Shehias with a *S. haematobium* prevalence of ≥ 3% in schoolchildren and/or ≥ 2% in community members were considered as hotspot IUs and received an intervention package consisting of (i) annual preventive chemotherapy with praziquantel in schools and communities; (ii) focal snail control with the molluscicide niclosamide; and (iii) BCC interventions. Shehias with a *S. haematobium* prevalence below the aforementioned thresholds were considered low-prevalence IUs and received surveillance-response interventions that included targeted treatment and focal snail control measures but did not include any BCC interventions.

### Cross-sectional surveys

The cross-sectional surveys for this study were conducted in the 16 main public primary schools in the study area. The baseline survey was carried out in November 2020 and the follow-up survey in March 2022. *S. haematobium* infection and schistosomiasis-related KAP were assessed in children attending grades 3, 4 or 5. In each grade, 25 children were selected; hence, 75 children per school [20]. For random selection, all children in a grade lined up, stratified by sex, and each third child, alternatively from the male and female rows, was systematically selected to be included in the study until 25 children per grade were reached [20].

Selected children received an information sheet and informed consent form (ICF) for their parents to sign. On the next day, when children had submitted the ICF signed by their parents or legal guardians, they were registered electronically by assigning them a unique identifier code (ID) and recoding their socio-demographic information (sex and age) using computer tablets (Samsung Galaxy Tab A; Samsung Electronics, Seoul, the Republic of Korea, 2019) and Open Data Kit (ODK, www.opendatakit.org) software.

Subsequently, each child was given a plastic container labelled with their ID and invited to provide its own urine sample. Urine samples were produced between 10 am and 2 pm and collected by the members of the study team who also interviewed the children about their schistosomiasis-related KAP. For this purpose, each child sat down in private with a study team member who asked questions in Kiswahili and recorded responses using a pre-tested ODK questionnaire (Additional file 1). The questions focused on children’s KAP regarding schistosomiasis transmission and prevention. There were questions about children’s knowledge of the lifecycle of *S. haematobium*, ways to prevent both getting infected with and spreading *S. haematobium* infection, their attitude towards schistosomiasis prevention of transmission and infection, and their access to improved water sources, their water contact behaviour and play practices.

### Laboratory procedures

Urine samples were transferred to the Public Health Laboratory-Ivo de Carneri (PHL-IdC) in Chake Chake on the day of collection. Urine samples were examined for *S. haematobium* eggs using a urine filtration method and microscopy by experienced laboratory technicians [26]. Data of the egg counts were recorded on paper sheets by laboratory technicians and subsequently double-entered into a Microsoft Excel database version 2016 (Microsoft Cooperation; Redmond Washington, USA) by two data entry clerks from PHL-IdC.

### BCC interventions

The BCC interventions in the SchistoBreak project were implemented from May to October 2021 in the five shehias that were stratified as hotspot IUs, based on the results of the first cross-sectional survey. The BCC tools and strategies used in the hotspot IUs in the SchistoBreak project area were originally developed in the ZEST project [17–19]. The measures were identified and designed together with the local population in Zanzibar, using a human-centred process, and included community-based and school-based interventions [28]. We adopted the following BCC measures that had worked well in the ZEST project and improved children’s knowledge and behaviour [29].

In communities, small-scale WASH measures (i.e. washing platforms) were installed in close proximity to a pump, tap or well. For this purpose, the behaviour study team organised meetings with community leaders (shehas) and community members to discuss the purpose and placement of the washing platform. Once an appropriate location was identified, the washing platforms were constructed with the labour support of the communities. The prerequisite for the location was that a working public tap or well was nearby or could be included directly into the platform. The round concrete platforms had a diameter of approximately 4 m, a sitting wall at the edge, and a drainage system with a slope where water could drain effectively into a large draining hole. Two washing platforms per hotspot IU were installed. Moreover, additional community meetings were held after construction to explain the advantages of using washing platforms for the prevention of schistosomiasis to the communities.

In schools, classroom- and school-based BCC interventions were implemented. For this purpose, teachers of all public primary schools and madrassas of the five hotspot IUs were invited for training at PHL-IdC. In the training, teachers learned about the transmission, prevention and symptoms of urogenital schistosomiasis, and methods for classroom-based participatory teaching, such as the application of blood fluke pictures, creating snail boards with students and joint drawing of *S. haematobium* life cycles [28]. Moreover, they practised approaches and safe play methods with educational messages about urogenital schistosomiasis for the implementation of school-based “Kichocho” (Kiswahili word for schistosomiasis) outreach days. After the training, the teachers were provided with blood fluke pictures, with educational flipcharts detailing the life cycle of *S. haematobium* and information about prevention of transmission and infection, with snail boards and with equipment to conduct “Kichocho days”, such as ropes and balls. The teachers were encouraged to apply the participatory methods they had learned in their classes and asked for an appointment for a Kichocho day in their schools, which they organised together with the behaviour team from PHL-IdC. During the intervention period, Kichocho days were implemented in the main public primary school and all madrassas in each hotspot IU. Teachers and students, together with the behaviour team, created and practised songs, dramas and games with messages about *S. haematobium* transmission and prevention, which were then performed by the students on a Kichocho day, in front of all students and teachers of the school and additional visitors, such as parents and community members.

In the first intervention period in 2021, the following BCC interventions were implemented: Two washing platforms were constructed per hotspot IU within a period of 5–6 days. On average, three community meetings were held in each hotspot IU. In each hotspot IU, the behaviour team used 2 days to deliver books about schistosomiasis, educational flipcharts, blood fluke pictures and snail boards to school and madrassa teachers. On average, six Kichocho days were implemented in the primary schools and madrassas of each hotspot IU. The preparation for Kichocho days by the behaviour team required an average of 2.8 days per IU. A total of 7334 children and 419 adults participated in the 30 Kichocho days during the intervention period in 2021.

### Sample size calculations

Considering a 20% dropout resulting from non-consenting parents or students’ absenteeism or inability to produce a sufficiently large urine sample, we aimed for a final sample size of 60 children per school and 960 children in all 16 schools in each of the annual cross-sectional surveys.

Assuming a prevalence of “good KAP” of 67%, the prevalence could be estimated with a precision of 7.4% points [precision = one-half length of the 95% confidence interval (*CI*)]. The intra-cluster correlation coefficient assumed for the sample size calculation of this study was 0.1.

### Statistical analysis

The KAP data collected in ODK were transferred directly to a secure ODK server at the Swiss Tropical and Public Health Institute (Swiss TPH; Allschwil, Switzerland). The statistical software R versions 3.5.1−4.3.2 (R Foundation for Statistical Computing, Vienna, Austria) were used to clean the double-entered electronic records of the laboratory data. Any discrepancies between the entries were traced back to the original paper records and corrected accordingly. After cleaning, the KAP and laboratory data sets were merged and analysed using R version 4.3.2.

*S. haematobium* egg counts were stratified into light-intensity (1–49 eggs/10 ml of urine) or heavy-intensity (≥ 50 eggs/10 ml of urine) infection, based on WHO guidelines [30]. The results were reported as the percentage of schoolchildren with positive test results.

All our questions about knowledge and attitude were multiple-choice questions, where several responses could be provided per question. A wrong response was scored as 0, a reasonable response was scored as 0.5 and a correct response was scored as 1 (Additional file 2). Knowledge and attitude were graded based on the participant’s final score for the questions under the respective outcome variable.

Four questions pertained to the participant’s knowledge about the cause of *S. haematobium* infection, where and how it is transmitted and the animal serving as an intermediate host in the transmission. Based on the minimum and maximum total knowledge scores (0 and 8) an individual could obtain, using different intervals of scoring points as cut-offs, knowledge was reported as a single outcome, with the following stratifications: no knowledge (0), poor knowledge (0.5 to 2.5), moderate knowledge (3 to 5.5), and good knowledge (6 to 8).

There were two questions pertaining to the participant’s attitude and knowledge about behaviour to prevent *S. haematobium* infection and transmission. Based on the minimum and maximum attitude scores, using intervals of 1 scoring point as cut-offs, attitude was reported as a single outcome, with the following stratifications: poor attitude (0 to 1), moderate attitude (1.5 to 2.5) and good attitude (3 to 4).

Ten questions pertained to the participant’s reported risky and protective practices related to *S. haematobium* exposure and infection. Risky practices included the crossing of a waterbody or rice field when going to school or a farm, and using natural open waterbodies for specific washing practices or for playing. Protective practices included playing at home, in the village, football field or in the bush. Here, no scoring system was used, but percentages of the total number of schoolchildren who carried out these practices were calculated for each practice.

Descriptive statistics (proportions and means) were used to assess the difference in schistosomiasis-related KAP in schoolchildren who received BCC interventions versus schoolchildren who did not receive BCC interventions, both at baseline in late 2020 and at the 1-year follow-up in early 2022.

Boxplots were used to indicate the distribution of knowledge and attitude scores of students in hotspot and low-prevalence schools at baseline and follow-up. A linear mixed-effect model accounting for clustering within schools was employed to determine the difference in mean knowledge scores and mean attitude scores between children from hotspot and low-prevalence schools at follow-up.

## Results

### Study participants

**Fig. 1:**
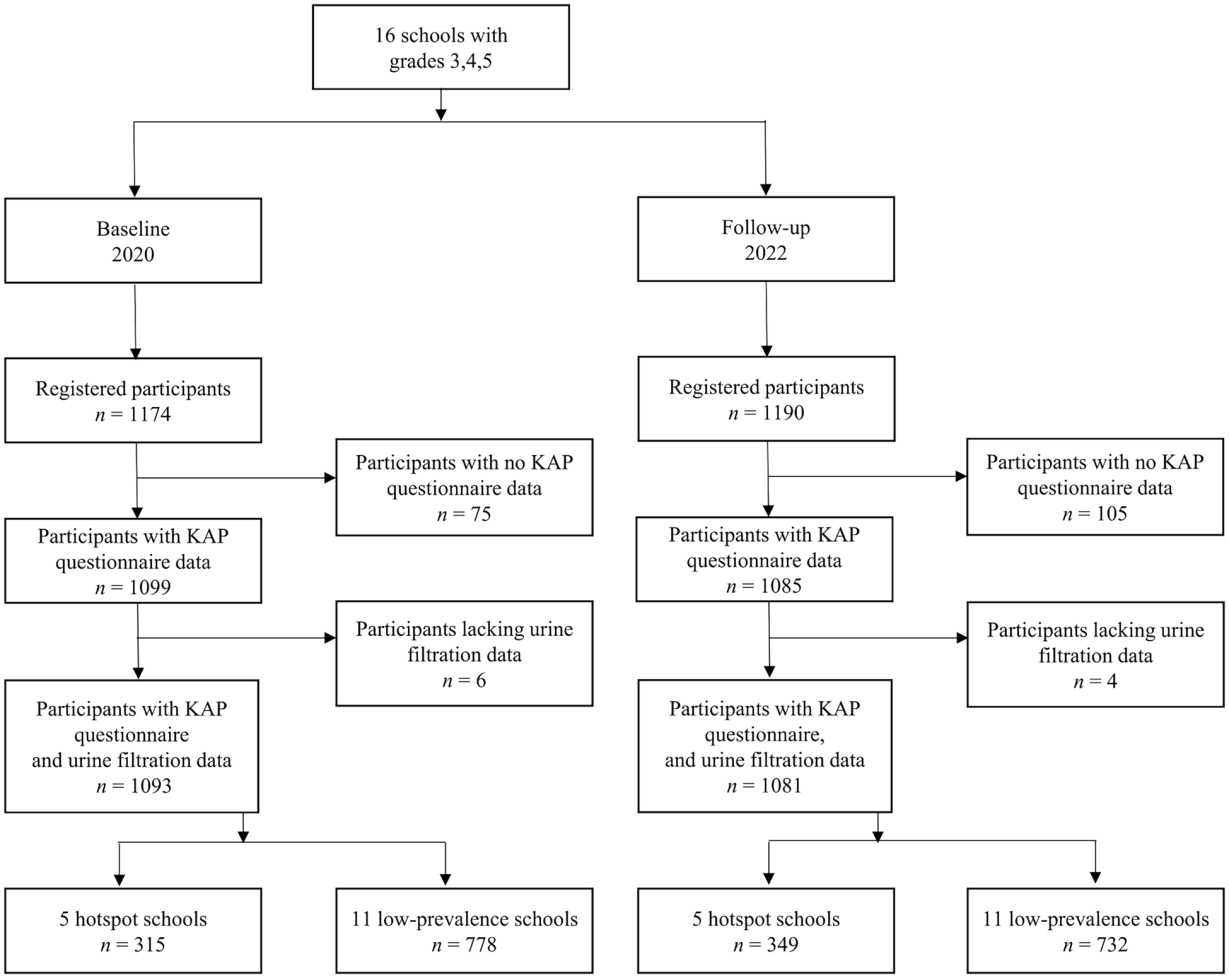
Study participation of schoolchildren in cross-sectional surveys in Pemba, Tanzania, at baseline and follow-up

In the 16 public primary schools, 1174 schoolchildren were registered to participate in the baseline survey in late 2020 and 1190 children were registered at follow-up in early 2022 (Fig. 1). Among them, 75 and 105 children, respectively, were excluded from the analyses as they had no KAP data, and six and four children, respectively, as they had no laboratory results. Taken together, KAP and laboratory data were available from 1093 children in late 2020 and 1081 children in early 2022. The participants belonged to the five hotspot schools (315 children in late 2020 and 349 children in early 2022) or the 11 low-prevalence schools (778 children in early 2020 and 732 children in early 2022).

### Characteristics of participating children

As indicated in Table 1, there were slightly more females than male participants in the baseline survey in late 2020 (583 vs 510). The median age of the children was 12 years (range: 8–16 years). There were similar numbers of children per grade (grade 3, *n* = 370; grade 4, *n* = 380; and grade 5,* n* = 343). At follow-up in early 2022, there were similar numbers of females and males (545 vs. 536). The median age was 11 years (range: 7–15 years). Children were equally repartitioned by grade (grade 3, *n* = 351; grade 4, *n* = 358; and grade 5, *n* = 372).

**Table 1:** Characteristics of schoolchildren in cross-sectional surveys in Pemba, Tanzania, at baseline and follow-up

Characteristic	Baseline (2020)						Follow-up (2022)					
	Total (*n* = 1093)		Hotspot (*n* = 315)		Low-prevalence (*n* = 778)		Total (*n* = 1081)		Hotspot (*n* = 349)		Low-prevalence (*n* = 732)	
	*n*	%	*n*	%	*n*	%	*n*	%	*n*	%	*n*	%
*Sex*												
Female	**583**	53.3	171	54.3	412	53.0	**545**	50.4	170	48.7	375	51.2
Male	**510**	46.7	144	45.7	366	47.0	**536**	49.6	179	51.3	357	48.8
*Age (years)*												
Minimum	8						7					
Maximum	16						15					
Median	12						11					
*Grade*												
Grade 3	**370**	33.9	111	35.2	259	33.3	**351**	32.5	113	32.4	238	32.5
Grade 4	**380**	34.7	113	35.9	267	34.3	**358**	33.1	118	33.8	240	32.8
Grade 5	**343**	31.4	91	28.9	252	32.4	**372**	34.4	118	33.8	254	34.7
*Schistosoma haematobium*	**19**	1.7	13	4.1	6	0.8	**12**	1.1	8	2.3	4	0.5

**Fig. 2:**
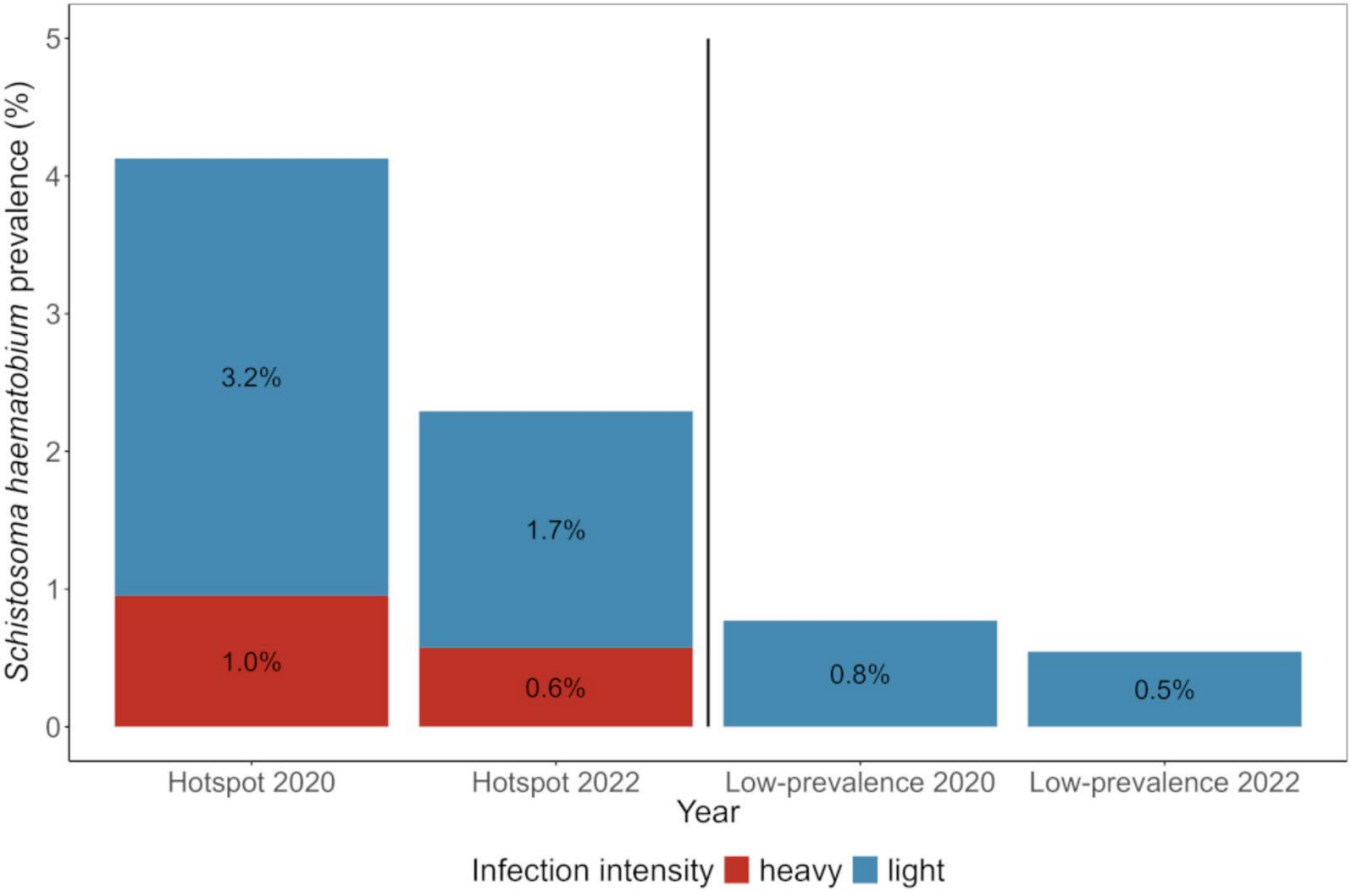
Prevalence of *Schistosoma haematobium* infection in children from hotspot and low-prevalence schools in Pemba, Tanzania, at baseline and follow-up

In the five hotspot schools, there were 13 (4.1%) *S. haematobium*-infected children at baseline in late 2020 and 8 (2.3%) at follow-up in early 2022 (Fig. 2). In the 11 low-prevalence schools, *S. haematobium* was detected in 6 (0.8%) children at baseline and 4 (0.5%) children at follow-up.

### Schoolchildren’s schistosomiasis-related knowledge and attitudes

**Fig. 3:**
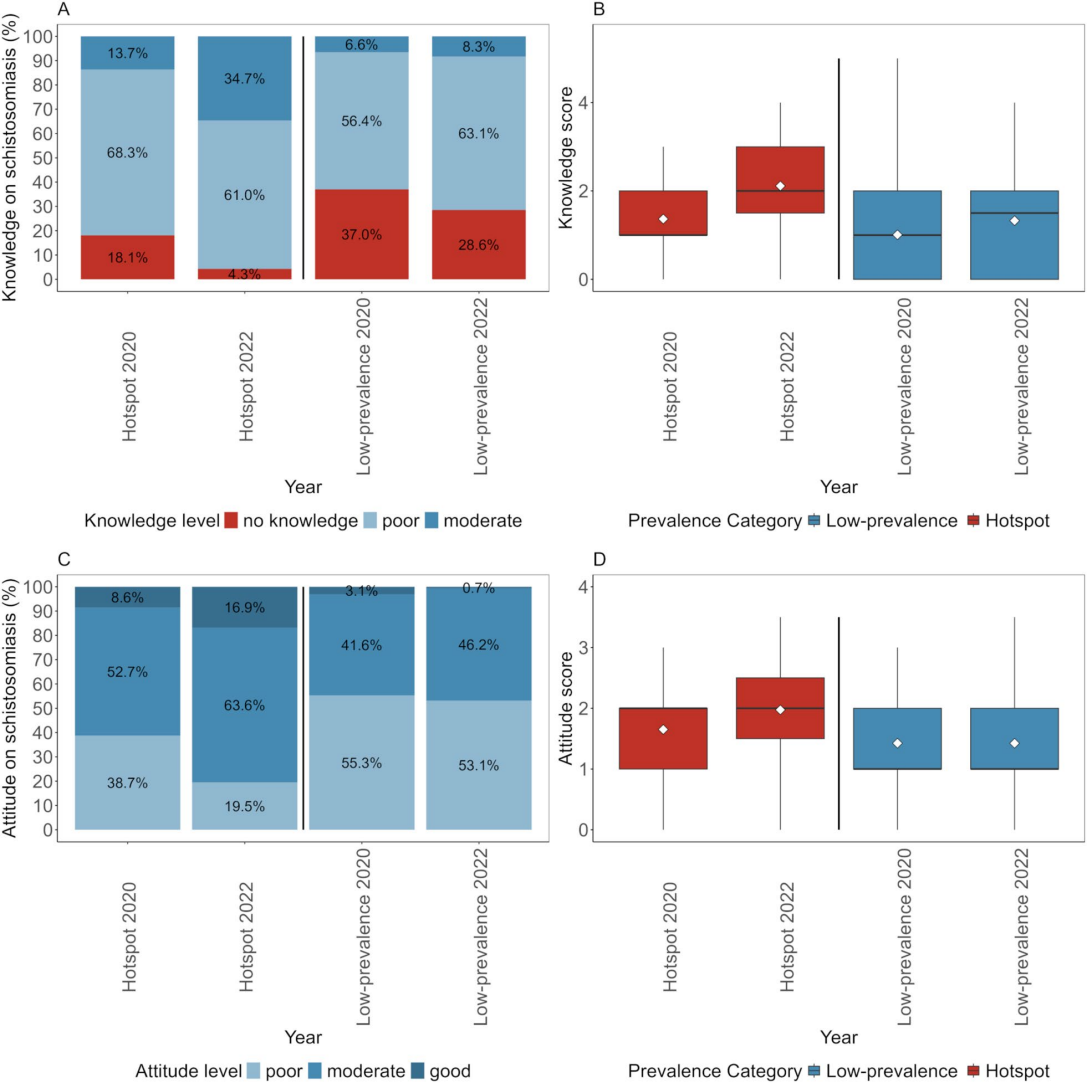
Schistosomiasis-related knowledge scores (**A**, **B**) and schistosomiasis-related attitude scores (**C**, **D**) in children from hotspot and low-prevalence schools in Pemba, Tanzania, at baseline and follow-up. The box plots (**B**, **D**) show min, max, median and interquartile ranges. The white diamond represents the arithmetic mean

In hotspot schools, 18.1% of the children had no knowledge about the cause and transmission of *S. haematobium* infection, 68.3% had poor knowledge and the remaining 13.7% had moderate knowledge at baseline in late 2020 (Fig. 3A). The schistosomiasis-related knowledge of children improved after the intervention period and 4.3% of the children had no knowledge, 61.0% had poor knowledge and 34.7% had moderate knowledge at follow-up in early 2022. Yet, none of the children achieved the scores for good knowledge.

In low-prevalence schools, where no BCC interventions were implemented, children had mostly no (37.0%) and poor (56.4%) knowledge at baseline in late 2020. Only a few children had moderate (6.6%) knowledge. The knowledge scores did not improve much at follow-up in early 2022, as students had no (28.6%), poor (63.1%) or moderate (8.3%) knowledge.

Overall, the mean knowledge scores of students from hotspot schools increased from 1.4 at baseline in late 2020 to 2.1 at follow-up in early 2022 (Fig. 3B). The mean knowledge scores of children from low-prevalence schools increased from 1.0 in 2020 to 1.3 in 2022. Comparing the mean knowledge scores between the settings, there was a statistically significant difference (0.8; 95% *CI*: 0.5−1.1) in the mean knowledge scores between children from hotspot and low-prevalence schools at follow-up.

In hotspot schools, the attitude of children represented in knowledge about how *S. haematobium* infection and transmission can be prevented, was mostly poor (38.7%) or moderate (52.7%) at baseline in late 2020 (Fig. 3C). The attitude improved after the 1-year BCC intervention period, fewer children scored with poor (19.5%) and more with moderate (63.6%) or good (16.9%) attitude. In the low-prevalence schools, the attitude of students remained similar at baseline in late 2020 and follow-up in early 2022 in all categories: poor (55.3% vs 53.1%), moderate (41.6% vs 46.2%) and good (3.1% vs 0.7%).

Overall, the mean attitude scores of students from hotspot schools increased from 1.7 at baseline in late 2020 to 2.0 at follow-up in early 2022 (Fig. 3D). The mean attitude scores of children from low-prevalence schools remained the same (1.4 in both 2020 and 2022). Comparing the mean attitude scores between the settings, there was a statistically significant difference (0.6; 95% *CI*: 0.4−0.7) between children from hotspot and low-prevalence schools at follow-up.

### Risky and preventive schistosomiasis-related practices of children

**Fig. 4:**
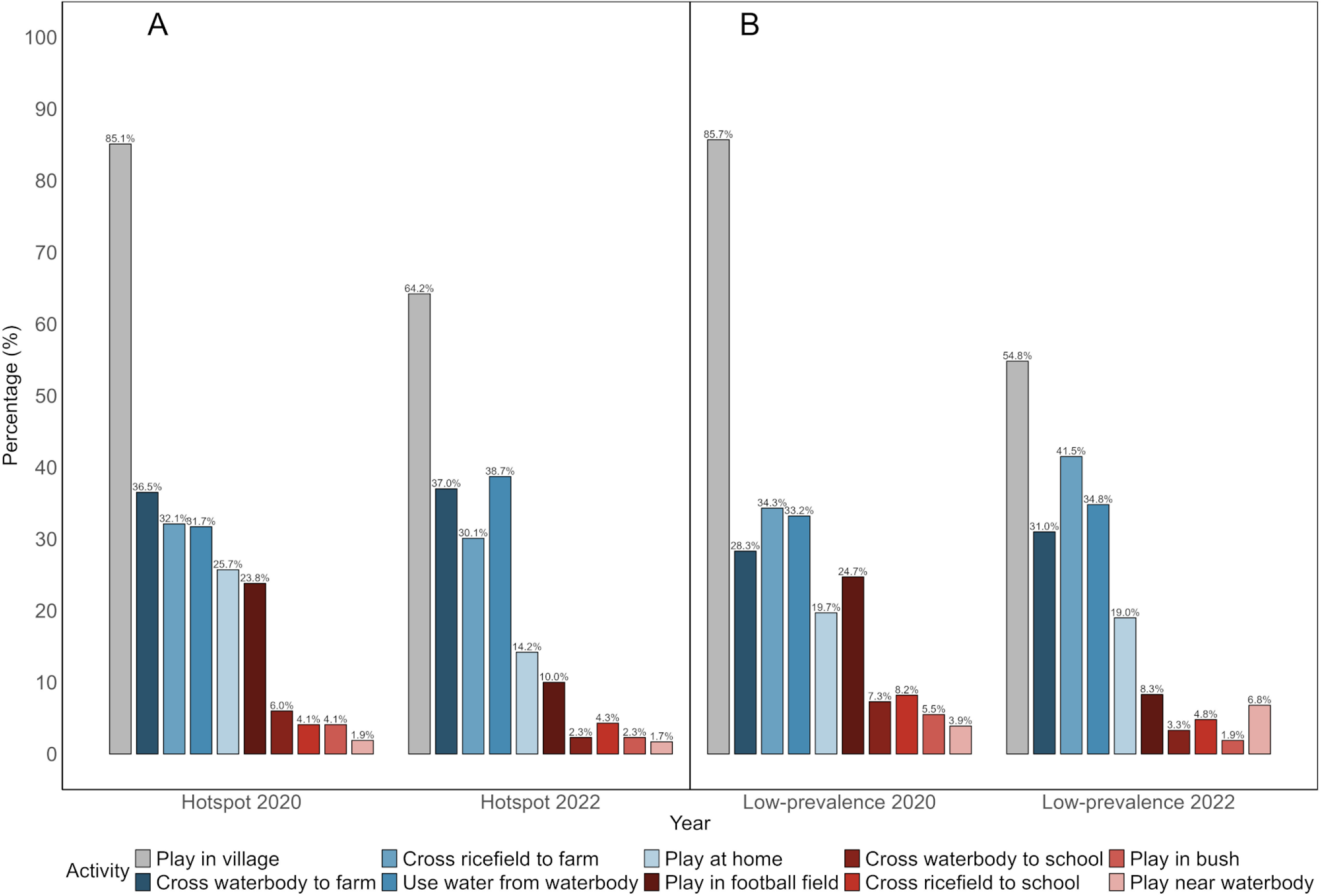
Risky and preventive schistosomiasis-related practices reported by children from hotspot (**A**) and low-prevalence (**B**) schools in Pemba, Tanzania, at baseline and follow-up

In hotspot schools, at baseline in late 2020, among 315 children who responded to the questionnaire, 56 (17.8%) children reported having a natural open waterbody near their homes, and 101 (32.1%) children reported using water from a natural open waterbody for different purposes (Fig. 4A). Furthermore, a total of 19 (6.0%) children reported crossing a waterbody on their way to school, 115 (36.5%) children reported crossing a waterbody on their way to a farm, 13 (4.1%) children reported crossing a rice field on their way to school, and 100 (31.7%) children reported to cross a rice field on their way to a farm. When asked with a multiple-choice option where they would mostly play, children reported playing in the village (85.1%), at home (25.7%), at the football field (23.8%) and in the bush (4.1%). Only a few children indicated that they would play in or near a natural waterbody (1.9%).

Similar responses were given at follow-up in early 2022. Among the 352 children with complete data records, 77 (21.9%) reported having a natural open waterbody in close proximity to their home, while 105 (29.8%) children reported using water from a natural open waterbody for any purpose. Eight (2.3%) children reported crossing a waterbody on their way to school, 129 (36.6%) children reported crossing a waterbody on their way to a farm, 15 (4.3%) children reported crossing a rice field on their way to school and 135 (38.4%) children reported to cross a rice field on their way to a farm. Children reported to play in the village (64.2%), at home (14.2%), at the football field (10.0%), in the bush (2.3%) or near a natural waterbody (1.7%).

In low-prevalence schools, at baseline in late 2020, among 778 students who responded to the questionnaire, 152 (19.5%) reported having a natural open waterbody in close proximity to their home and 267 (34.3%) children reported using water from a natural open waterbody for any purposes (Fig. 4B). Fifty-seven (7.3%) children reported crossing a waterbody on their way to school, 220 (28.3%) children reported crossing a waterbody on their way to a farm, 64 (8.2%) children reported crossing a rice field on their way to school and 258 (33.2%) children reported to cross a rice field on their way to a farm. Children reported playing in the village (85.7%), at home (19.7%), at the football field (24.7%), in the bush (5.5%) or near a natural waterbody (3.9%).

At follow-up in early 2022, among 733 students, 197 (26.9%) children reported having a natural open waterbody near their home, and 304 (41.5%) children reported using water from a natural open waterbody for any purpose. Twenty-four (3.3%) children reported crossing a waterbody on their way to school, 227 (31.0%) children reported crossing a waterbody on their way to a farm, 35 (4.8%) children reported crossing a rice field on their way to school and 255 (34.8%) children reported to cross a rice field on their way to a farm. Children reported to play in the village (54.8%), at home (19.0%), at the football field (8.3%), in the bush (1.9%) or near a natural waterbody (6.8%).

### Schoolchildren’s washing practices

**Fig. 5:**
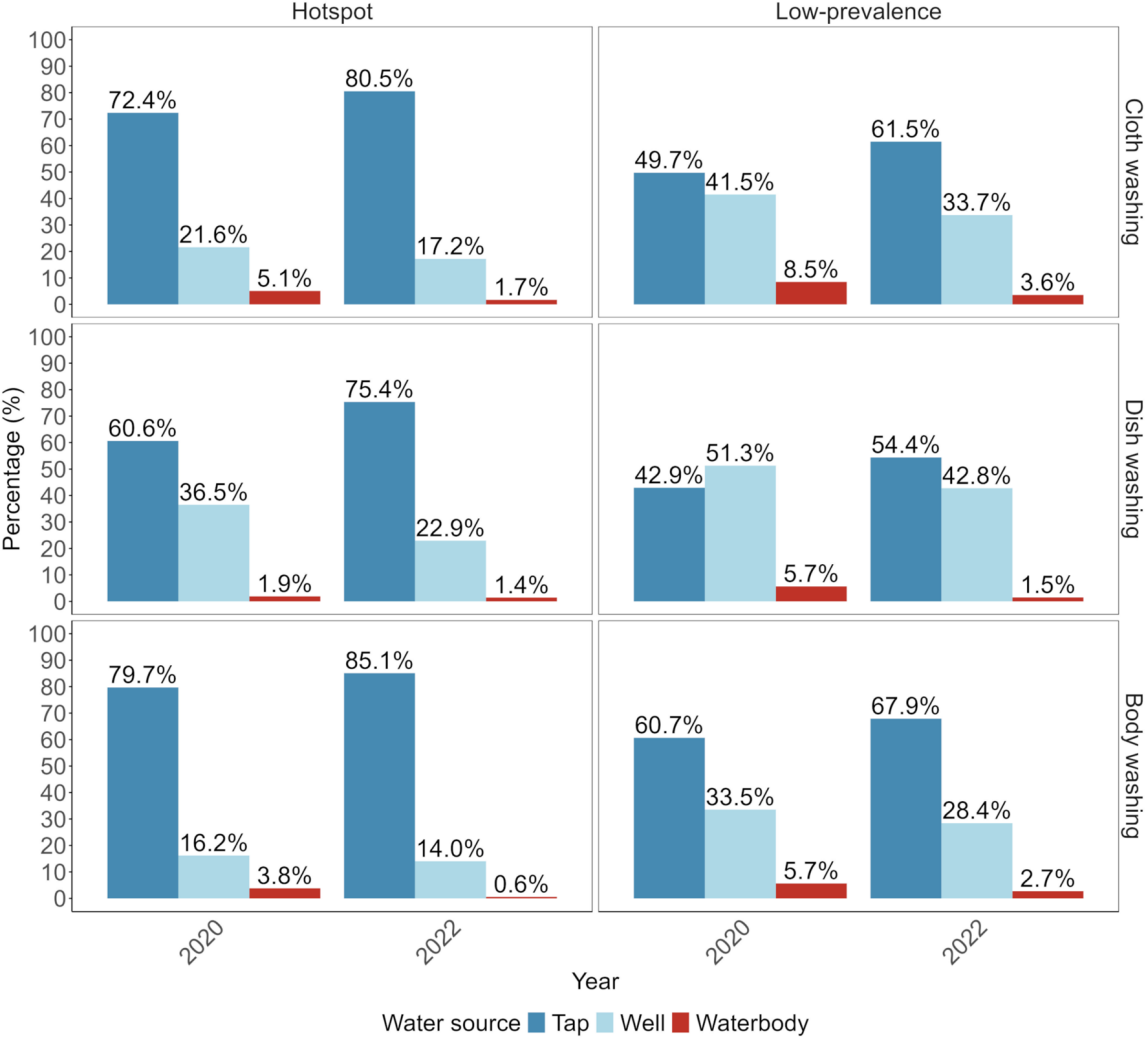
Water usage for washing practices by children from hotspot and low-prevalence schools in Pemba, Tanzania, at baseline and follow-up

In hotspot schools, both at baseline and follow-up, most children reported using a tap or well for washing clothes, dishes or their body (Fig. 5). Moreover, at baseline, 5.1%, 1.9% and 3.8% reported that they would also use water from a natural open waterbody for washing clothes, dishes or their body, respectively. At follow-up, the percentages decreased and only 1.7%, 1.4% and 0.6% of the children reported that they would use water from a natural open waterbody for washing clothes, dishes or their body, respectively.

In low-prevalence schools, mostly taps or wells were used for washing clothes and dishes and for bathing. However, at baseline, more children in low-prevalence than in hotspot schools reported that they would use water from a natural open waterbody for washing clothes (8.5%), dishes (5.7%) or their bodies (5.7%). At follow-up, also in low-prevalence schools, fewer children reported using water from a natural open waterbody for washing clothes (3.5%), dishes (1.5%) or their bodies (2.7%).

## Discussion

Over large parts of sub-Saharan Africa, including Pemba, considerable progress has been made in the control of schistosomiasis over the past 20 years. To further reduce transmission and achieve elimination, WHO considers BCC as well as WASH interventions, environmental management and focal snail control as essential complementary measures to preventive chemotherapy [2, 9]. Knowledge about the health consequences of schistosomiasis and how the disease can be prevented and transmission interrupted is vital for people living in endemic areas to change their attitudes and practices and, ultimately, their behaviour. We aimed to assess the 1-year impact of BCC interventions on schistosomiasis-related KAP of schoolchildren within the frame of the SchistoBreak project in Pemba [20].

We found that BCC interventions implemented in five hotspot schools had a positive impact on children’s knowledge about the cause and transmission of urogenital schistosomiasis. Our results are in line with those of a previous study conducted on the Zanzibar islands in 2017, which showed a significant increase in knowledge about *S. haematobium* of children who had received BCC interventions compared with children who had not received the interventions [29]. Moreover, children from Nigeria who participated in “Schisto and Ladders™” games with specific health education messages about schistosomiasis, had better knowledge about schistosomiasis and praziquantel treatment compared with their counterparts who did not play the game [31, 32]. In Mozambique, community members who received a community dialogue intervention, which aimed to strengthen the prevention and control of schistosomiasis, demonstrated improved knowledge about transmission and behaviour that put them at risk for schistosomiasis [33]. These findings indicate that dialogues with the community and participatory BCC interventions are important measures to improve knowledge about schistosomiasis, and hence, pave the way for changing behaviour to prevent infection and transmission.

Indeed, the BCC interventions in our study had a positive impact on children’s attitude towards *S. haematobium* infection and transmission. The knowledge about behaviour that can help to prevent infection and transmission, such as not playing in waterbodies or not urinating in the water, improved. Improvements in knowledge, perceptions and self-reported reduction of risky behaviours were also observed after the implementation of BCC interventions in Zanzibar and elsewhere in Tanzania and after health education interventions in Côte d’Ivoire and the People’s Republic of China [29, 34–36]. These findings emphasise that health education and active participatory BCC interventions can have a positive effect on the attitude towards schistosomiasis of children and other community members. Only if people understand the consequences of infection and disease and only if they understand how transmission occurs, preventive action may be taken. Encouraging the change of behaviour and a correct attitude, particularly of population groups that are at high risk of infection, are essential to achieving the ambitious schistosomiasis control and elimination goals by 2030.

Finally, a decrease in the use of natural open waterbodies for washing and other practices was detected in children from schools that received or did not receive BCC interventions. Hence, the change in this risky practice cannot directly be associated with the BCC interventions but may also be an effect caused by changing environmental factors, seasonality or the annual precipitation and related availability of waterbodies. It may also be that the result is a spillover effect, when children from low-prevalence schools learned from friends visiting a hotspot school or when they visited a madrassa in a hotspot area, where BCC interventions were applied. Moreover, children from low-prevalence schools were exposed to surveillance-response interventions that may have created an awareness of *Schistosoma* transmission in natural open waterbodies and a subsequent change in behaviour. Other studies from Zanzibar showed that children improved their schistosomiasis-related behaviour after receiving health education or BCC, respectively [29, 37]. A study from Mozambique, where community members had received community dialogue interventions, reported a considerable increase in protective behavioural practices against schistosomiasis [33]. Examples of improvements in protective behaviour due to educational measures also exist for other diseases. For example, in Côte d’Ivoire, behaviour to prevent soil-transmitted helminth and intestinal protozoa infections improved once an integrated intervention package, including community-led total sanitation and health education, was implemented [34]. In Ghana, improved uptake of preventive measures against malaria by women was reported after the implementation of BCC [38].

Importantly, as with other interventions, BCC needs to be implemented with a high coverage and long-term to be effective. Many studies have discussed the relevance of using strategies with a wide population reach for schistosomiasis control programmes in endemic settings [17, 39–42]. With the Kichocho day outreach events in our study, several thousand children and other community members were reached and learned about urogenital schistosomiasis in an interactive and engaging way. Schools are an ideal venue for BCC to control and eliminate schistosomiasis since many individuals can be reached in one go and school-aged children are at highest risk of *Schistosoma* infections [14, 16, 43]. Students with good schistosomiasis-related knowledge may serve as important behaviour-change agents and role models by transmitting their knowledge to siblings, parents and other community members. Hence, the continued exploration of BCC interventions alongside other recommended interventions could be a strong force in strengthening schistosomiasis control and elimination programmes in endemic settings [44]. The impact of long(er)-term BCC and the sustainability of KAP after cessation of BCC interventions will be explored in future analyses of the SchistoBreak project [45].

To achieve a successful change in behaviour, not only does the KAP of people need to be improved, but WASH infrastructure needs to become available that allows the implementation of preventive practices. The washing platforms that were constructed in close proximity to improved water sources in hotspot shehias of our study area were a step in this direction. They already were considered a safe and attractive alternative to washing in natural open waterbodies where people might acquire a *S. haematobium* infection in the ZEST project [15, 29], and they were widely used in the SchistoBreak project. In addition, the implementation of sanitation infrastructure such as latrines may support a behaviour that prevents *Schistosoma* transmission [46–48]. However, the effect of latrines may be more profound for *S. mansoni*, which is transmitted via faeces. *S. haematobium*, which is the only autochthonous species infecting humans in Zanzibar [5, 49, 50], is transmitted via urine. Stopping people from urinating into open water bodies is difficult, since many feel an urge to urinate when they get in contact with water, even if they went to toilet before. Therefore, and since the latrines constructed during the ZEST project were not frequently used and rapidly fell into disrepair [16], we decided in the SchistoBreak project to prioritize the implementation of washing platforms and not to install latrines.

To enable large-scale change of behaviours, the communities themselves, governments, and/or external funders will need to invest in improved water supplies and sanitation. Community ownership and acceptance will be key for their sustained use and maintenance [40, 51]. Only if people understand the importance of using clean water and have access to a reliable supply, the transmission of *S. haematobium* can be prevented and elimination can be achieved and sustained. Hence, a close collaboration between the health, education, water and sanitation sectors and the communities is warranted for ultimate success [5].

There are several limitations of this study, which may have biased our results. First, efforts to control and eliminate urogenital schistosomiasis from Zanzibar are ongoing since decades and the whole population has been exposed to regular treatment and awareness campaigns. Hence, our study population was not “naïve” in their knowledge about schistosomiasis. Second, as described above, some schoolchildren who attended a low-prevalence school may live in a hotspot area or visit a madrassa where they were exposed to BCC interventions, resulting in a spillover effect. Finally, the single BCC intervention period assessed here was short and the number of BCC interventions children from hotspot schools received was very limited. Since the acquisition of knowledge and a related change in behaviour takes time and needs repeated exposure to BCC interventions, longer-term studies are needed to assess the full impact of BCC interventions.

## Conclusions

The BCC interventions implemented in the SchistoBreak project effectively improved the schistosomiasis-related KAP of schoolchildren in the North of Pemba. Well-designed and longer-term BCC interventions that are adapted to local cultures and needs, and are created and implemented in close collaboration with local communities, have the potential to advance schistosomiasis control and elimination programmes by raising awareness and allowing schoolchildren and community members to take practical actions to prevent infection and spreading. However, a durable change in behaviour can only occur if access to clean water and sanitation is improved. Hence, intersectoral collaboration and community empowerment are important ingredients for eliminating schistosomiasis as a public health problem and breaking transmission in Pemba and other areas that aim to reach the goals put forth in the WHO roadmap towards 2030.

## Supplementary Information

Additional file 1. Questionnaire to assess the schistosomiasis-related knowledge, attitude and practices of children.

Additional file 2. System to score the responses to questions pertaining to schistosomiasis-related knowledge and attitudes of children.

## Data Availability

The datasets used and/or analysed during the current study are available from the corresponding author upon reasonable request.

## Abbreviations

BCC: Behaviour change communication
*CI*: Confidence interval
ICF: Informed consent form
ID: Identification
IU: Intervention unit
KAP: Knowledge, attitude and practices
ODK: Open Data Kit
WASH: Water, sanitation and hygiene
WHO: World Health Organization
