# Supplementary material for: One-year impact of behavioural interventions on schistosomiasis-related knowledge, attitude and practices of primary schoolchildren in Pemba, Tanzania
Source: Infect Dis Poverty. 2024 Nov 13;13:84. doi: 10.1186/s40249-024-01251-y (PMC11558867; doi:10.1186/s40249-024-01251-y)
Supplement: Supplementary file 1 — Additional file 1. Questionnaire to assess the schistosomiasis-related knowledge, attitude and practices of children. [file 40249_2024_1251_MOESM1_ESM.pdf]

Additional file 1:

Questions assessed for the manuscript “One-year impact of behavioural interventions on schistosomiasis-related knowledge, attitude and practices amongst primary schoolchildren in Pemba, Tanzania” by Ndum et al. are highlighted in grey.

Questionnaire for to assess schoolchildren’s knowledge, attitude and practices related to schistosomiasis

District: |__________________| Shehia: |__________________________________| **Participant ID __ __--__ __ __ __--__ __--__ __ __**

Name of school: |__________________________________|

Interviewer Name: ____ Date of interview (DD/MM/YYYY): |__|__|/|__|__|/|__|__|__|__|

**Demographics**		
	1.	Name: _______________________________________
	2.	Sex (M/F): _________
	3	Grade: _________ Grade letter: _________
	4	How old are you (in years)? ________
	5	Shehia of residency name: _______________________________________
**Schistosomiasis transmission**		
	6	Have you ever heard about schistosomiasis? ____no ___yes ____ I do not know
	7	If yes, where did you hear about schistosomiasis? ____School ____Radio ____TV ____Drug distributor ____I do not know ____ Other
	8	If other, specify_________________________
	9	What do you think is the cause of schistosomiasis? ____Blood fluke ____Worm ____I do not know …____Other

	10	If other, specify___________________________
	11	Where do you think schistosomiasis is transmitted? ____Rice field ____Toilet ____River/pond ____Trash/dump ____I do not know ____Other
	12	If other, specify___________________________
	13	During which activities do you think you get schistosomiasis? ____Playing with sand ____Playing in dirty water ____Playing in the river/pond ____Swimming in the river or pond ____Washing with river water ____Fishing from river water ____Farming with river water ____Walking barefoot ____I do not know ____Other
	14	If other, specify___________________________
	15	Do you know which animal is needed for the transmission of schistosomiasis? ____Blood fluke ____Worm ____Snail ____Bug ____I do not know ____Other
	16	If other, specify___________________________
**Schistosomiasis prevention**		
	17	Which behavior(s) can help to NOT get infected with schistosomiasis? ____Not playing in the river/pond ____Not wash in river/pond ____Not swimming in river/pond ____Use tap water/well water ____Play somewhere else than in the river/pond ____I do not know ____Other
	18	If other, specify___________________________
	19	Which behavior(s) can help to NOT transmit schistosomiasis? ____Not to urinate into river/pond ____Take treatment ____I do not know ____ Other
	20	If other, specify___________________________
**Water use**		
	21	What kind of water do you usually use for washing clothes? ____River/pond ____Tap ____Well ____I do not know ____ Other
	22	If other, specify___________________________
	23	What kind of water do you usually use for washing the dishes? ____River/pond ____Tap ____Well ____I do not know ____ Other
	24	If other, specify___________________________

	25	What kind of water do you usually use for washing your body? ____River/pond ____Tap ____Well ____I do not know ____ Other
	26	If other, specify__________________________
**River use**		
	27	Is there a river/stream or pond/lake nearby your home? ____no ___yes ____ I do not know
	28	Do you sometimes use water from the river/pond for any other purpose? ____no ___yes ____ I do not know
	29	If yes, for what purpose? __________________________
**Schistosomiasis education at school**		
	30	Has your teacher taught you about schistosomiasis? ____no ___yes ____ I do not know
	31	When did your teacher talk last about schistosomiasis? ____This term ____Last year ____Long ago ____I do not know
	32	What kind of teaching tools was the teacher using? ____Snail board ____Flipchart ____Blood fluke picture ____No tools ___I do not know ____Other
	33	If other, specify__________________________
	33	Have you ever attended a Kichocho Day at school? ____no ___yes ____ I do not know
	35	When did you attend a Kichocho Day for the last time? ____This term ___Last year ____Long ago ____ I do not know
	36	Which activity of the Kichocho day did you like most? ____Poems ___ Games ____Drama ____ Songs ____Education ___I do not know ____Other
	37	If other, specify__________________________
**Risk factors**		
	38	Where do you typically play? ____At home (inside the house) ___ In the village (outside of the house) ____ In the forest ____ In the river/pond ____ In the bush ___Football field ___ I do not know ___ Other
	39	If other, specify__________________________

	40	Do you sometimes play in the river/pond? ____no ___yes ____ I do not know
	41	Do you cross a river/pond when you go to school? ____no ___yes ____ I do not know
	42	Do you cross a river/pond when you go to shamba? ____no ___yes ____ I do not know
	43	Do you cross a ricefield when you go to school? ____no ___yes ____ I do not know
	44	Do you cross a ricefield when you go to shamba? ____no ___yes ____ I do not know
**Laundry platforms**		
	45	Are you aware of any laundry platform installed by the kichocho team in your shehia? ____no ___yes ____ I do not know
	46	Are you using the laundry platform in your shehia? ____no ___yes ____ I do not know
	47	How often are you using the laundry platform? ____Everyday ____At least once per week ____Less than once per week ____Less than once per month ____I do not know
**Treatment**		
	48	Have you received treatment against schistosomiasis in the last 6months? ____no ___yes ____ I do not know
	49	If yes, where have you received treatment against schistosomiasis? ____MDA: at home ____MDA: in school ____T&T: at a waterbody by test-and-treat ____T&T: in school ____In a hospital ____In a health facility ____I do not know ____Other
	50	If other, specify___________________________
	51	Were you measured for your height to receive this drug? ____no ___yes ____ I do not know
	52	Were you tested for schistosomiasis before you got this drug? ____no ___yes ____ I do not know
	53	If yes, how many tablets of praziquantel did you receive? ____ 0 ____0.5 ____1 ____1.5 ____2 ____2.5 ____3 ____More than 3 ____I do not know
	54	If yes, how many tablets of praziquantel did you swallow? ____ 0 ____0.5 ____1 ____1.5 ____2 ____2.5 ____3 ____More than 3 ____I do not know
	55	Did you throw up after swallowing the drugs? ____no ___yes ____ I do not know
