# Supplementary material for: One-year impact of behavioural interventions on schistosomiasis-related knowledge, attitude and practices of primary schoolchildren in Pemba, Tanzania
Source: Infect Dis Poverty. 2024 Nov 13;13:84. doi: 10.1186/s40249-024-01251-y (PMC11558867; doi:10.1186/s40249-024-01251-y)
Supplement: Supplementary file 2 — Additional file 2. System to score the responses to questions pertaining to schistosomiasis-related knowledge and attitudes of children. [file 40249_2024_1251_MOESM2_ESM.pdf]

Additional file 2:

System to score the responses to questions pertaining to schistosomiasis-related knowledge and practices of children in the manuscript “One-year impact of behavioural interventions on schistosomiasis-related knowledge, attitude and practices amongst primary schoolchildren in Pemba, Tanzania” by Ndum et al.

Question number	Question	Response option	Score
**Knowledge**			
1	What do you think is the cause of schistosomiasis?	Blood fluke	1
		Worm	0.5
		Other	0
		I do not know	0
1a	What do you think is the cause of schistosomiasis? (other)	Snail	1
		Swimming in rivers/lakes	0.5
		Bacteria	0
		Go to the toilet	0
		Disposing of dirty water	0
		Swimming in dirty water	0
		Flies	0
		Flies and dirty water	0
		To eat dirty food	0
		Dirt	0
		Other unrelated responses	0
2	Where do you think schistosomiasis is transmitted?	River/pond	1
		Ricefield	1
		River/pond and toilet	0.5

		Ricefield and trash/dump	0.5
		River/pond and trash/dump	0.5
		Trash/dump	0
		Toilet	0
		I do not know	0
		Other	0
2a	Where do you think schistosomiasis is transmitted? (other)	In pools of water	0.5
		In the urine	0.5
		In dirty water	0
		In the sea	0
		At home	0
		At dirty places	0
		In the bush	0
		In the hospital	0
		In the desert	0
		At the well	0
		Other unrelated responses	0
3	During which activities do you think you get schistosomiasis?	Playing in the river/pond	1
		Swimming in the river/pond	1
		Washing with river water	1
		Fishing from river water	1
		Farming with river water	1
		Playing in the river/pond and walking barefoot	0.5
		Washing with river water and playing with sand	0.5
		Playing with sand	0
		Playing in dirty water	0
		Walking barefoot	0

		I do not know	0
		Other	0
3a	During which activities do you think you get schistosomiasis? (other)	Bathing in the river	1
		Crossing through the river	1
		Passing through rice fields	1
		Urinating in water sources (river/ponds)	0.5
		Playing in the valley	0
		Playing on the ropes	0
		Playing cards	0
		Playing unsafe games	0
		Other unrelated responses	0
4	Do you know which animal is needed for transmission of schistosomiasis?	Blood fluke	1
		Snail	1
		Bug	0
		Worm	0
		I do not know	0
		Other	0
4a	Do you know which animal is needed for transmission of schistosomiasis? (other)	*Schistosoma*	1
		Malaria	0
		Eggs of insects	0
		Mosquito	0
		Other unrelated responses	0
**Attitude**			
1	Which behaviour(s) can help to NOT get infected with schistosomiasis?	Not playing in the river/pond	1
		Not wash in river/pond	1
		Not swimming in river/pond	1
		Play somewhere else than in the river/pond	1
		Use tap water/well water	0.5

		I do not know	0
		Other	0
1a	Which behaviour(s) can help to NOT get infected with schistosomiasis? (other)	Playing safe games	1
		Protect yourself from playing with dirty water	1
		Treat river	1
		Wear shoes, use the toilet	0.5
		Do not bathe in dirty water	0.5
		Do not play with dirty water	0.5
		Not eating unwashed fruits	0
		Body hygiene	0
		Do not play with dangerous substances	0
		Do not go to the valley	0
		Avoid playing in the sand	0
		Other unrelated responses	0
2	Which behaviour (s) can help to NOT transmit schistosomiasis?	Not to urinate into river/pond	1
		Take treatment	1
		I do not know	0
		Other	0
2a	Which behaviour (s) can help to NOT transmit schistosomiasis? (other)	Urinating in the toilet	1
		To prevent people from going to the ponds	1
		Go to the hospital	1
		Do not play with water	1
		Do not go to rivers	1
		All schistosomiasis patients should be treated	1
		Not swimming in pools	1
		Wear shoes, use the toilet	0.5
		Do not swim in rivers/ponds	0.5
		Drink clean water	0

		Clean the toilet after urinating	0
		Not donating blood	0
		Use clean water	0
		Do not hang out with other people	0
		Do not stay with your partner	0
		Sleep alone	0
		Not shaking hands	0
		Other unrelated responses	0
